# Automatic anatomical classification of esophagogastroduodenoscopy images using deep convolutional neural networks

**DOI:** 10.1038/s41598-018-25842-6

**Published:** 2018-05-14

**Authors:** Hirotoshi Takiyama, Tsuyoshi Ozawa, Soichiro Ishihara, Mitsuhiro Fujishiro, Satoki Shichijo, Shuhei Nomura, Motoi Miura, Tomohiro Tada

**Affiliations:** 1 Tada Tomohiro Institute of Gastroenterology and Proctology, Saitama, Japan; 2 Department of Surgical Oncology, Graduate School of Medicine, The University of Tokyo, Tokyo, Japan; 3 Department of surgery, Sanno Hospital, International University of Health and Welfare, Tokyo, Japan; 4 Department of Gastroenterology, Graduate School of Medicine, the University of Tokyo, Tokyo, Japan; 5 Department of Gastrointestinal Oncology, Osaka International Cancer Institute, Osaka, Japan; 6 Department of Global Health Policy, Graduate School of Medicine, The University of Tokyo, Tokyo, Japan; 7 Department of Epidemiology and Biostatistics, School of Public Health, Imperial College London, London, United Kingdom; 8 Graduate School of Public Health, Teikyo University, Tokyo, Japan

## Abstract

The use of convolutional neural networks (CNNs) has dramatically advanced our ability to recognize images with machine learning methods. We aimed to construct a CNN that could recognize the anatomical location of esophagogastroduodenoscopy (EGD) images in an appropriate manner. A CNN-based diagnostic program was constructed based on GoogLeNet architecture, and was trained with 27,335 EGD images that were categorized into four major anatomical locations (larynx, esophagus, stomach and duodenum) and three subsequent sub-classifications for stomach images (upper, middle, and lower regions). The performance of the CNN was evaluated in an independent validation set of 17,081 EGD images by drawing receiver operating characteristics (ROC) curves and calculating the area under the curves (AUCs). ROC curves showed high performance of the trained CNN to classify the anatomical location of EGD images with AUCs of 1.00 for larynx and esophagus images, and 0.99 for stomach and duodenum images. Furthermore, the trained CNN could recognize specific anatomical locations within the stomach, with AUCs of 0.99 for the upper, middle, and lower stomach. In conclusion, the trained CNN showed robust performance in its ability to recognize the anatomical location of EGD images, highlighting its significant potential for future application as a computer-aided EGD diagnostic system.

## Introduction

Over the decade, remarkable progress has been made in the field of computational image recognition. Prior to this, the computational analysis of images was based mainly on feature quantities defined by humans, such as color, brightness, shape, textural pattern and other distinguishing features. However, this type of analysis is limited by image rotation, the lack of brightness, adjacent or angular views of the object, or blurring of the image^1,2^. Recently, the mainstream architecture of computational image recognition has been gradually replaced by deep learning convolutional neural networks (CNNs)^3,4^. This technique takes advantage of multiple network layers (consecutive convolutional layers followed by pooling layers) to extract key features from an image and output a final classification through the fully connected layers. The most impactful feature of this deep learning method is self-learning; once a training data set has been provided, the program can extract key features and quantities without any human indication by using a back-propagation algorithm and by changing the internal parameters of each neural network layer.

This methodology has been applied to a variety of medical fields in an effort to develop computer-aided systems that can support the diagnosis of physicians. Previous reports have shown that CNN technology performed at a high level when applied to radiological diagnosis^5–8^, skin cancer classification^9^ and diabetic retinopathy^10^. Furthermore, CNN was reported to be highly beneficial in the field of endoscopy, by assisting with the diagnosis of capsule video images, and detecting and diagnosing colorectal polyps^11–13^.

Esophagogastroduodenoscopy (EGD) is the main modality used in the diagnosis of upper gastrointestinal diseases, such as reflux esophagitis, gastroduodenal ulcer, and gastric cancer^14,15^. Although EGD is a widely-performed technique, physicians require specific training because EGD specialists must be equipped with not only the appropriate techniques also sufficient knowledge to identify and diagnose these diseases properly because of subtle changes or physiological resemblance with other diseases^16^. Therefore, in this respect, a CNN system which has been trained to support the diagnosis of gastrointestinal diseases may help to reduce the burden upon physicians. In order for CNN systems to diagnose gastrointestinal diseases, the first crucial step is for the system to properly recognize the anatomical location. This is important because images of the GIE can include four main different organs: the larynx, esophagus, stomach, and the duodenum. Furthermore, there are distinct characteristics of the normal anatomical structure of the stomach when compared between the upper, middle, and lower regions. Without learning these normal anatomical features, it is very difficult to recognize abnormalities and to diagnose diseases properly^15,16^. Therefore, in the present study, we aimed to construct a CNN system that could correctly identify the normal anatomical location as a preliminary step for the future development of a computer-aided EGD diagnostic system. We constructed a CNN-based diagnostic program using a large data set of 27,335 EGD images from 1,750 patients and validated the performance of this CNN using 17,081 independent EGD images from 435 patients. To the best of our knowledge, this is the first study to construct and evaluate a computer-aided diagnostic system using CNN in the field of EGD. Our CNN showed robust performance in recognizing the anatomical position of EGD images, thus highlighting the significant potential for the future application of this CNN as a computer-aided EGD diagnostic system.

## Results

### The newly-developed CNN recognized the main anatomical location of EGD images with high accuracy

First, EGD images were divided into four main categories (larynx, esophagus, stomach and duodenum). Our newly-developed CNN classified the anatomical location correctly for 16,632 (97%) out of 17,081 images. The PS, which the CNN calculated, ranged from 0–100%, and the individual highest PSs of 96% of the images were more than 90% (Table 1).

**Table 1:** The distribution of the probability score and the accuracy of the CNN system.

Probability Score	Correct number (%)	Whole number (%)	Accuracy (%)
>99%	15,168 (91)	15,265 (89)	99.4
99–90%	980 (6)	1,101 (6)	89.0
90–70%	336 (2)	437 (3)	76.9
70–50%	143 (1)	264 (2)	54.2
<50%	5 (0)	14 (0)	35.7
Total	16,632 (100)	17,081 (100)	97.4

**Figure 1:**
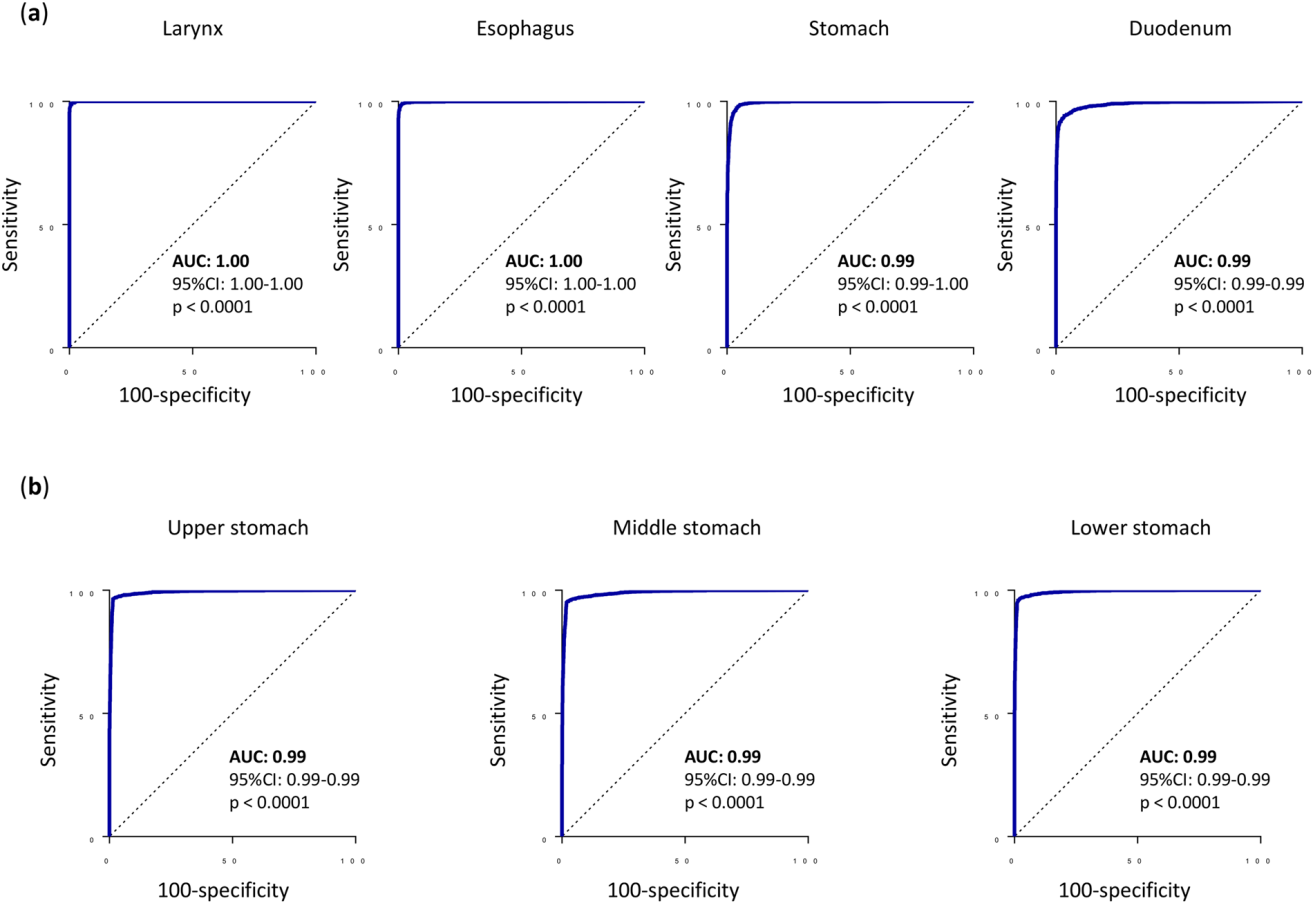
(**a**) The convolutional neural network (CNN) recognized the anatomical location of gastro-intestinal endoscopy images very accurately with area under the curve (AUC) values of 1.00 [95% confidence interval (CI): 1.00-1.00, p < 0.0001] for the larynx, 1.00 (95%CI: 1.00–1.00, p < 0.0001) for the esophagus, 0.99 (95%CI: 0.99–1.00, p < 0.0001) for the stomach, and 0.99 (95%CI: 0.99–0.99, p < 0.0001). (**b**) The convolutional neural network (CNN) recognized the anatomical location of the stomach images accurately with AUC values of 0.99 (95%CI: 0.99–0.99, p < 0.0001) for the upper stomach, 0.99 (95%CI: 0.99–0.99, p < 0.0001) for the middle stomach, and 0.99 (95%CI: 0.99-0.99, p < 0.0001) for the lower stomach.

Our CNN recognized the anatomical location of the GIE images accurately with AUC values of 1.00 [95% confidence interval (CI): 1.00–1.00, p < 0.0001] for larynx, 1.00 (95%CI: 1.00–1.00, p < 0.0001) for esophagus, 0.99 (95%CI: 0.99–1.00, p < 0.0001) for stomach, and 0.99 (95%CI: 0.99–0.99, p < 0.0001) for duodenum, as shown in Fig. 1a.

The sensitivity and specificity of the CNN for recognizing the anatomical location of each category were: 93.9% and 100% for larynx; 95.8% and 99.7% for esophagus; 98.9% and 93.0% for stomach; and 87.0% and 99.2% for duodenum (Table 2).

**Table 2:** Anatomical classification of the images of gastrointestinal scope by CNN.

Output	Larynx (n = 363) (%)	Esophagus (n = 2,142) (%)	Stomach (n = 13,048) (%)	Duodenum (n = 1,528) (%)
Larynx	341 (94)	3 (0)	1 (0)	0 (0)
Esophagus	5 (1)	2,053 (96)	28 (0)	9 (1)
Stomach	17 (5)	75 (4)	12,908 (99)	189 (12)
Duodenum	0 (0)	11 (1)	111 (1)	1,330 (87)
Sensitivity (%)	93.9	95.8	98.9	87.0
Specificity (%)	100	99.7	93.0	99.2

### The CNN correctly classified EGD images of the stomach into three anatomical locations (upper, middle, and lower stomach)

It is important to determine the anatomical location in the stomach because some gastric diseases tend to occur in specific areas of the stomach. Therefore, we were curious as to whether the CNN could correctly recognize the anatomical location of images acquired from different parts of the stomach.

The validation set featured a total of 13,048 stomach images, including 3,532 images from the upper stomach, 6,379 images from the middle stomach and 3,137 images from the lower stomach. The CNN recognized the anatomical location of these stomach images accurately with AUC values of 0.99 (95%CI: 0.99–0.99, p < 0.0001) for the upper stomach, middle stomach, and lower stomach, as shown in Fig. 1b.

The sensitivity and specificity of the CNN in recognizing the anatomical location of each category were: 96.9% and 98.5% for the upper stomach, 95.9% and 98.0% for the middle stomach, and 96.0% and 98.8% for the lower stomach (Table 3).

**Table 3:** Anatomical classification of stomach images by CNN.

Output	Upper (n = 3,532) (%)	Middle (n = 6,379) (%)	Lower (n = 3,137) (%)
Upper	3,423 (97)	148 (2)	15 (0)
Middle	60 (2)	6,119 (96)	75 (2)
Lower	8 (0)	32 (1)	3,012 (96)
The others	41 (1)	80 (1)	35 (1)
Sensitivity (%)	96.9	95.9	96.0
Specificity (%)	98.5	98.0	98.8

### Evaluation of images which were incorrectly classified

**Figure 2:**
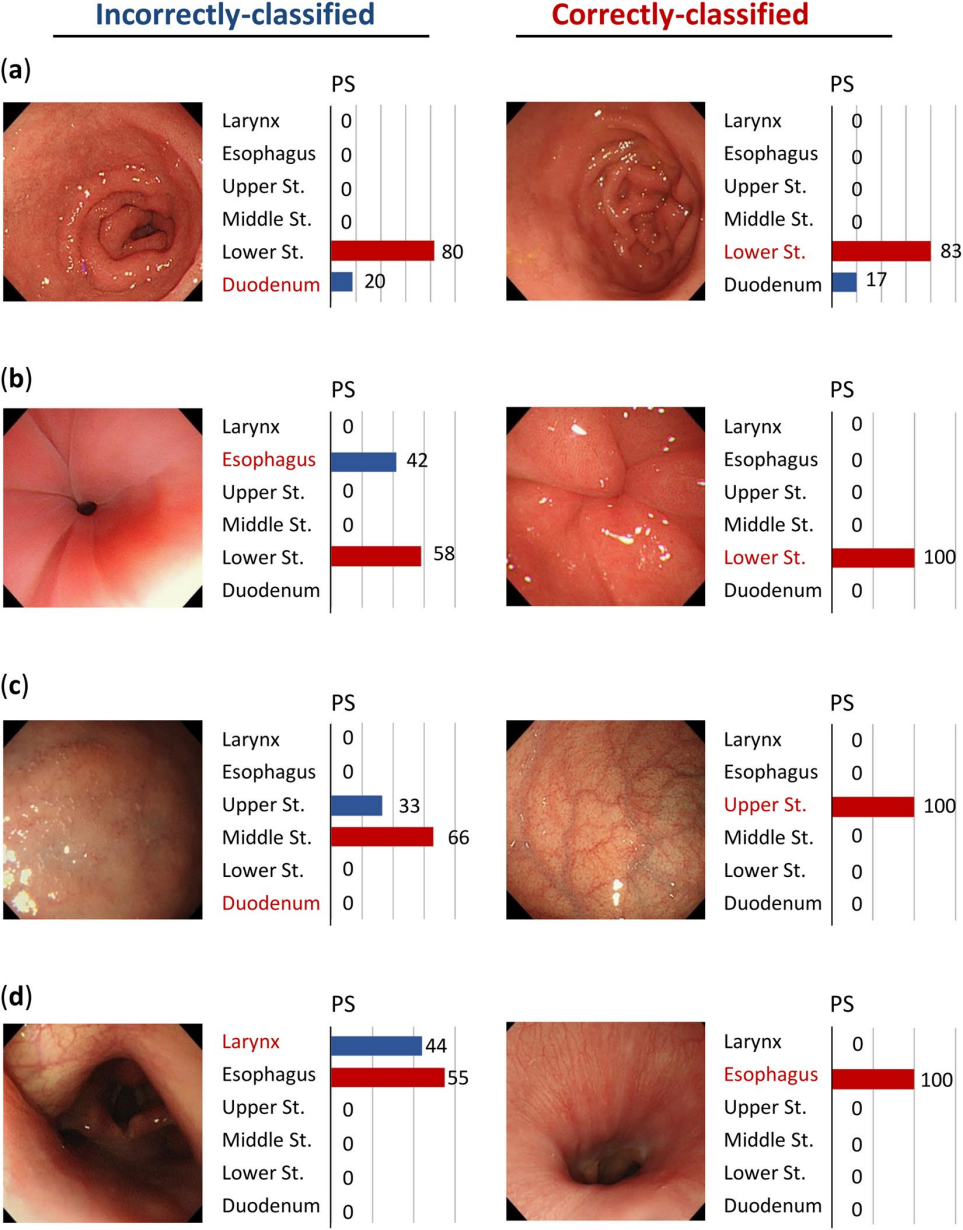
Examples of images which were correctly and incorrectly classified by the convolutional neural network (CNN). PS represents the probability score. (**a**) Duodenum image, which was incorrectly classified by the CNN as the lower stomach (left) and a correctly classified image from the duodenum (right). (**b**) Esophagus image, which was incorrectly classified by the CNN as the lower stomach (left) and a correctly classified image from the lower stomach (right). (**c**) Duodenum image, which was incorrectly classified by the CNN as the middle stomach (left) and a correctly classified image from the upper stomach (right). (**d**) Larynx image, which was incorrectly classified by the CNN as the esophagus (left) and a correctly classified image from the esophagus (right).

Finally, we reviewed images which had been incorrectly classified by the CNN in order to provide a basis for improving the performance of the CNN. Typical examples of incorrectly classified images are shown in Fig. 2a–d.

Figure 2a shows an image of the duodenum which was miss-classified as the lower stomach. As seen in these images, in landscape view, the duodenum sometimes shows a resemblance to the lower stomach; we believe that this was the cause of the incorrect classification. Figure 2b,c show images of the esophagus and duodenum which were miss-classified as the lower stomach and the upper stomach, respectively. In these images, gross structure could not be used for recognition due to insufficient insufflation of the lumen and/or the proximity of the scope to the luminal wall. On the other hand, Fig. 2d shows an image of the larynx, which was apparently different in its gross appearance, and was incorrectly classifies as the esophagus.

## Discussion

Architecture for the classification of images is constantly advancing^4^. Using such technology, the classification error rate was reported 3.0–7.3%, according to the latest results of the Imagenet Large Scale Visual Recognition Challenge 2016 (ILSVRC2016, http://image-net.org/challenges/LSVRC/2016/results). Our newly-developed CNN system showed a high accuracy (97%) with high AUCs of 0.99–1.00, thus demonstrating the significant potential of CNN in the classification of EGD images according to anatomical location. This ability to recognize anatomical classification was an important step in clarifying whether CNN systems could be used in the diagnosis of gastrointestinal diseases by detecting lesions or abnormal findings automatically on images acquired from patients. The next step will be to evaluate whether such systems can be used to diagnose different diseases and features, such as polyps, ulcers or cancers. Because physicians need significant training, over several years, to become specialists of EGD, this system could help to reduce their burden and also be beneficial for patients. In this regard, the results of this study showed promising potential for the CNN to establish a novel EGD support system. Furthermore, automatic anatomical classification of the endoscopic images can itself be beneficial in the clinical setting, because EGD images, which are randomly collected and stored electronically, are often reviewed by a second observer in order not to miss diseases; anatomically classified images are easier for such personnel to interpret and will thus reduce their burden.

The methodology required in order to obtain further accuracy remains unclear. For example, images can be compromised by bleeding (especially after biopsy), bubbles, partial blurring, partial defocusing or endoscopic devices such as forceps. However, these factors did not seem to affect the output from our CNN much, since the accuracy for images affected by these problems remained almost the same as those which were unaffected, even we deleted these images from training data (data not shown), although highly compromised images could not be evaluated correctly. Physicians classify EGD images using a combination of gross structure and surface texture. Sometimes, our CNN incorrectly classified images; we suspect that this occurred because even though gross structure was evident, there was a resemblance to the gross structure of other organs, as suggested in Fig. 2a. This type of incorrect classification may be reduced by increasing the number of training images and by acquiring further information about surface structure and texture. In some images, structure proved to be difficult to recognize, mainly because the images were taken closely to the surface, or were insufficiently insufflated by air, as shown in Fig. 2b,c. In such cases, it might be necessary to teach the CNN about information of the surface microstructure, such as those which can be acquired by magnified dye endoscopy^17^ or new generation endoscopy that may be available in near future, such as the 4 K or 8k ultra-high definition system^18^. Furthermore, some images, which captured characteristic organ structure, were incorrectly classified in a way that endoscopists would never miss, as shown in Fig. 2d. Although the precise reason for this remains unclear, this type of incorrect classification might be due to the limited ability of the CNN used in the current study, which will be more sophisticated in future.

Intrinsically, the anatomical location of human organs should be recognized under a three-dimensional spatial position. Since an image created by computed tomography (CT) scanning merely represents an axial slice of a target organ, anatomical organ recognition with CNN using CT images has already been shown to be successful^5,7^. The cross-sectional surface of a given organ could be described in almost the same manner by CT in each case. On the other hand, images obtained by endoscopy represented an overhead view of the lumen and therefore varied between cases in terms of form, length, width and breadth. Even for the same case, images vary, even when taken by the same endoscopist, because of the viewing angle, closeness to the surface, or the expansiveness of the organ following insufflation. This matter might be resolved in future by introducing another form of architecture, such as panoramic mapping using endoscopic video images^19,20^. Furthermore, training the CNN with high quality images featuring more segmental categories could also lead to more accurate results.

There are several limitations to this study which need to be considered. Firstly, this study was conducted using images from single institute and it is therefore uncertain whether our CNN system will work in the same way using other image sets because the architecture of the CNN or the internal parameters or weights of each layer were invisible. Secondly, all our images were processed only through Olympus Medical Systems, and test images were not processed by any other supplier. Therefore, we are now conducting a multi-institutional study to construct and validate a much more powerful CNN system for EGD.

In conclusion, our trained CNN showed robust performance in the classification of EGD images based upon their anatomical location thus highlighting its significant potential for future application as a computer-aided EGD diagnostic system.

## Materials and Methods

### Esophagogastroduodenoscopy procedures

A total of 33 endoscopists performed EGD at Tada Tomohiro Institute of Gastroenterology and Proctology, Saitama, Japan, from January 2014 to March 2017. Indications for EGD were referral from a primary care physician concerning current epigastric symptoms, positive results from gastric disease screening by barium contrast examination or abnormal serum pepsinogen levels, a previous history of gastro-duodenal disease, or simple screening.

GIE images were taken using standard endoscopy (GIF-XP290N, GIF-XP260, GIF-XP260NS, GIF-N260; Olympus Medical Systems, Co., Ltd., Tokyo, Japan) and a standard endoscopic system (EVIS LUCERA ELITE; Olympus Medical Systems, Co., Ltd., Tokyo, Japan). All endoscopists were instructed to take whole pictures of the larynx, esophagus, stomach, and duodenum, even if there were no abnormalities. The typical number of images taken for a patient without gastrointestinal disease was 34 (larynx 1, esophagus 6, stomach 25, duodenum 2).

### Image preparation for training set

More than 70,000 EGD images were collected from 1,750 patients between January 2014 and December 2016; these were reviewed by gastroenterologists. Images were excluded if they focused on diseases in which it was difficult to recognize the anatomical location, or which were not clear because of food residue, bleeding, halation, blur or poor focus. The remaining 27,335 images were categorized into six main categories (larynx, esophagus, duodenum, and upper, middle, lower stomach). Each of the main stomach categories were comprised of a number of sub-categories; the upper stomach consisted of the cardia and upper body, the middle stomach consisted of the angle and the middle body, while the lower stomach consisted of the pylorus, antrum, and lower body (Table 4). These 27,335 original endoscopic images were randomly rotated between 0 and 359 degrees, the black frame surrounding each image was cropped on each side and software used to either zoom in or out by a factor of 0.9 or 1.1. Finally, the number of training images was amplified to in excess of 1 million. Only regular white light images, with normal magnification, were included and enhanced images, such as narrow band images, were excluded from the training set. All accompanying patient information was annotated prior to data analysis, and none of the endoscopists involved in the study were able to access any identifiable patient information. This study was approved by the Institutional Review Board of Japan Medical Association (ID: JMA-IIA00283).

**Table 4:** Anatomical classification of the training image set and validation image set.

Main category	Sub category	Training set (n) (%)	Validation set (n) (%)
Larynx		663 (2)	363 (2)
Esophagus	Upper, middle part	1,460 (5)	2,142 (13)
	Lower part	1,792 (7)	
Upper stomach	Cardia	1,830 (7)	3,532 (21)
	Upper body	3,649 (13)	
Middle stomach	Angle	2,247 (8)	6,379 (37)
	Middle body	4,937 (18)	
Lower stomach	Antrum	2,517 (9)	3,137 (18)
	Pylorus	3,012 (11)	
	Lower body	2,010 (7)	
Duodenum	Bulbs	1,578 (6)	1,528 (9)
	Second portion	1,640 (6)	
Total		27,335 (100)	17,081 (100)

### Image preparation for the validation set

In order to evaluate the diagnostic accuracy of the CNN, an independent set of 17,081 images from 435 consecutive patients receiving endoscopic examination at Tada Tomohiro Institute of Gastroenterology and Proctology between February 2017 and March 2017 were collected and prepared as a validation image set. Only regular white light images with normal magnification were included. Enhanced images, or images in which the anatomical location could not be recognized, were excluded from the validation set in the same manner as for the training set. Finally, the validation set featured 17,081 images, including 363 larynx images, 2,142 esophageal images, 13,048 stomach images, and 1,528 duodenal images (Table 4).

### CNN algorithm

The CNN algorithm was developed using GoogLeNet (https://arxiv.org/abs/1409.4842), a deep neural network architecture with 22 layers. We then used the Caffe deep learning framework, one of the most popular and widely used frameworks, originally developed at the Berkeley Vision and Learning Center (BVLC), to train, validate and test our newly-developed CNN. Our deep CNN was trained using back-propagation, a method of training neural networks, in which gradients for all weights in the network can be computed efficiently. All layers of the network were fine-tuned using Adam (https://arxiv.org/abs/1412.6980), a method for stochastic optimization, with a global learning rate of 0.0002. To ensure that all our images were compatible with GoogLeNet, we resized each image to 244 × 244 pixels.

The trained CNN system created a probability score (PS) for each image ranging from 0 to 100% which shows the probability for a given image belonging to each of the anatomical classifications. The category with the highest PS was adopted as the final classification for the CNN, and the CNN-classified images were then evaluated manually by two authors (HT and TT) with specialist knowledge of gastroenterology to assess whether they had been correctly classified or not. If the diagnosis differed between the two doctors, then discussion was used to arrive at a satisfactory resolution.

### Statistics

**Figure 3:**
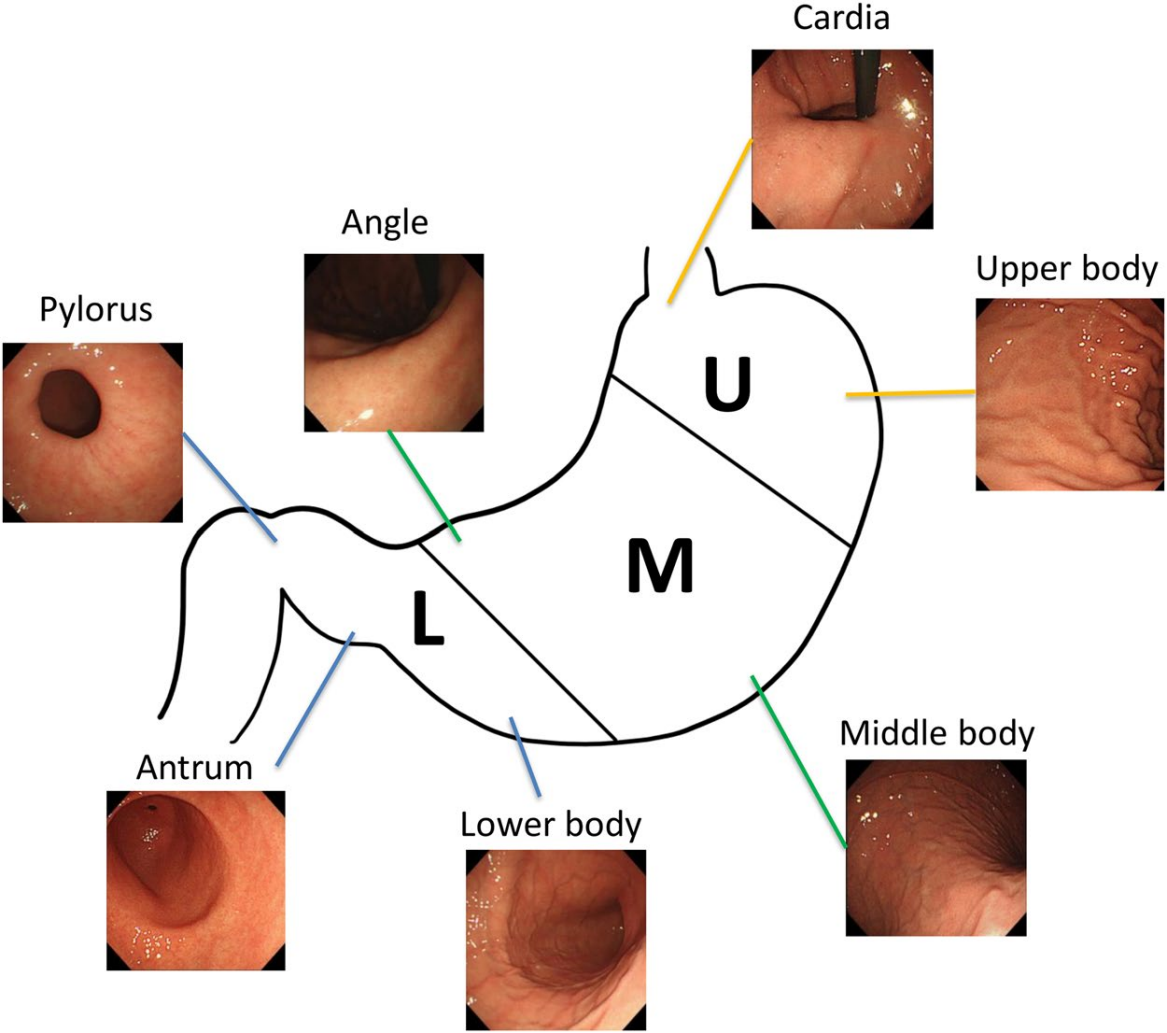
The main anatomical categories and sub-anatomical categories of the stomach according to Japanese guidelines.

**Figure 4:**
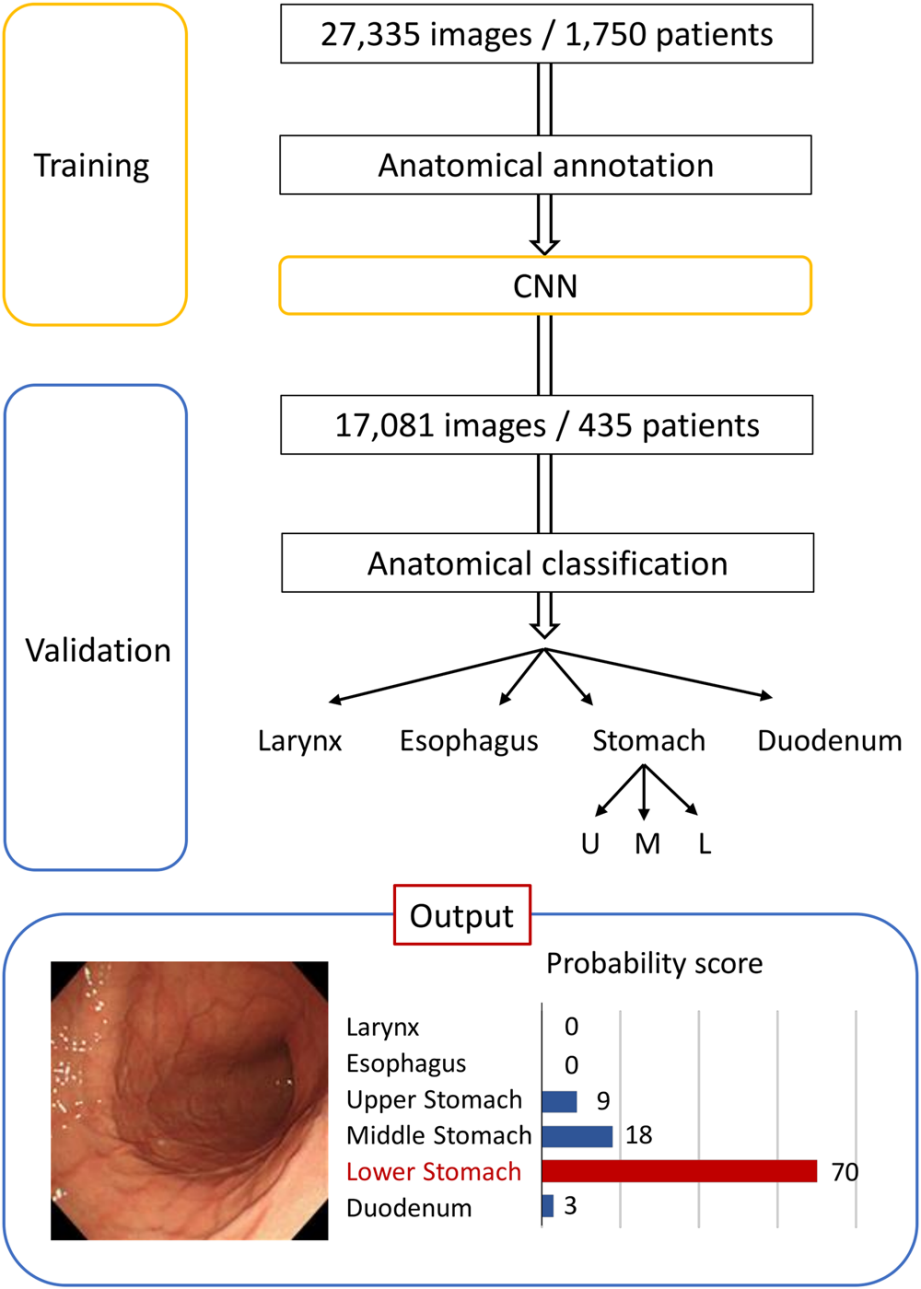
A flow chart depicting our study design. The lower figure shows a representative image of the output probability score using a convolutional neural network (CNN) for gastro-intestinal images (in this case, an image from the lower stomach).

We evaluated two main end-points; firstly, whether the CNN could recognize images from four different organs (larynx, esophagus, stomach, and duodenum) and secondly, whether the CNN could recognize the stomach location (upper, middle, lower stomach) according to the Japanese Classification of Gastric Carcinoma (3rd English edition)^21^, as shown in Fig. 3. Sensitivity and specificity were calculated for the CNN anatomical classifications. Receiver operating characteristic (ROC) curves were plotted for the classification of each organ, and the area under the curves (AUCs) were calculated by GraphPad Prism 7 (GraphPad software, Inc, California, U.S.A.). A brief summary of the study design is shown in Fig. 4.
